# Interim estimates of vaccine effectiveness against influenza A(H1N1)pdm09 and A(H3N2) during a delayed influenza season, Canada, 2024/25

**DOI:** 10.2807/1560-7917.ES.2025.30.4.2500059

**Published:** 2025-01-30

**Authors:** Lea Separovic, Yuping Zhan, Samantha E Kaweski, Suzana Sabaiduc, Sara Carazo, Romy Olsha, Richard G Mather, James A Dickinson, Maan Hasso, Isabelle Meunier, Agatha N Jassem, Nathan Zelyas, Ruimin Gao, Nathalie Bastien, Danuta M Skowronski

**Affiliations:** 1 British Columbia Centre for Disease Control, Vancouver, Canada; 2 Institut National de Santé Publique du Québec, Québec, Canada; 3 Public Health Ontario, Toronto, Canada; 4 Queen’s University, Kingston, Canada; 5 University of Calgary, Calgary, Canada; 6 Public Health Laboratory, Alberta Precision Laboratories, Edmonton, Canada; 7 National Microbiology Laboratory, Public Health Agency of Canada, Winnipeg, Canada; 8 University of British Columbia, Vancouver, Canada

**Keywords:** influenza, vaccine effectiveness, test-negative design, A(H1N1)pdm09, A(H3N2)

## Abstract

The Canadian Sentinel Practitioner Surveillance Network (SPSN) reports interim 2024/25 vaccine effectiveness (VE) against acute respiratory illness due to laboratory-confirmed influenza during a delayed season of predominant A(H1N1)pdm09 and lower A(H3N2) co-circulation. Through mid-January, the risk of outpatient illness due to influenza A is reduced by about half among vaccinated vs unvaccinated individuals. Adjusted VE is 53% (95% CI: 36–65) against A(H1N1)pdm09, comprised of clades 5a.2a and 5a.2a.1, and 54% (95% CI: 29–70) against A(H3N2), virtually all clade 2a.3a.1.

The 2024/25 influenza season in Canada has thus far been characterised by delayed season onset and, similar to 2023/24, predominant A(H1N1)pdm09 circulation with lesser A(H3N2) contribution [1]. Co-circulation of A(H1N1)pdm09 clades 5a.2a and 5a.2a.1 continues alongside A(H3N2) clade 2a.3a.1. The Canadian Sentinel Practitioner Surveillance Network (SPSN) reports interim 2024/25 vaccine effectiveness (VE) against influenza A(H1N1)pdm09 and A(H3N2), with whole genome sequencing of case viruses for context.

## Epidemiological context

Vaccine effectiveness was assessed using a test-negative design (TND). Community-based sentinel practitioners in SPSN provinces – Alberta, British Columbia (BC), Ontario and Quebec – collected nasal/nasopharyngeal specimens from consenting patients presenting with acute respiratory illness (ARI; new or worsening cough potentially due to infection) within 7 days of illness onset. Specimens were tested by accredited provincial laboratories using real-time RT-PCR and/or multiplex assays. Influenza vaccine status was determined by participant or guardian report. This mid-season analysis includes specimens collected between 27 October 2024 (epi-week 44) and 18 January 2025 (epi-week 3) from eligible patients aged ≥ 1 year old. To address possible bias related to correlated COVID-19 and influenza vaccination behaviour, SARS-CoV-2-positive individuals were excluded from influenza controls in VE sensitivity analyses [2]. Sparse data issues were addressed with Firth’s penalised logistic regression [3].

Most publicly funded vaccines in SPSN provinces were inactivated (99%) and egg-based (90%). Cell-based vaccines were used in Alberta (< 50%; targeted to those aged 6 months to 64 years) and Ontario (< 10%; authorised for ≥ 6 months old). In 2024/25, only the A(H3N2) vaccine component was updated, now a clade 2a.3a.1 strain (from A/Darwin/9/2021 to A/Thailand/8/2022 in egg-based vaccines; from A/Darwin/6/2021 to A/Massachusetts/18/2022 in cell-based vaccines) [4]. The A(H1N1)pdm09 component, belonging to clade 5a.2a.1, remains unchanged from 2023/24 (A/Victoria/4897/2022 in egg-based; A/Wisconsin/67/2022 in cell-based) [4]. Influenza B components also remain unchanged [4]. Community-dwelling adults aged ≥ 65 years (≥ 75 years in Quebec) were administered high-dose (Alberta, Ontario, Quebec) or adjuvanted (BC, Ontario, Quebec) influenza vaccines.

## Virological characterisation

We attempted whole genome sequencing of as many influenza A case viruses as possible up to the time of publication. This was undertaken by Canada’s National Microbiology Laboratory for context and interpretation of VE findings [5]. The HA clades and subclades were assigned as per Nextclade [6], specifying amino acid substitutions and affected antigenic sites in parentheses, and annotating receptor binding site (RBS) involvement and gain/loss of glycosylation, e.g. +/− CHO, all in relation to vaccine reference strains available in GISAID as listed in Supplementary Table S1 [7].

## Virological findings

Within the SPSN network, 2024/25 influenza activity has been relatively delayed compared with recent prior seasonal analyses and is most comparable to the 2015/16 A(H1N1)pdm09 epidemic [8-10]. Of 4,459 eligible specimens, 647 (15%) have been influenza-positive to date, reaching 5% in week 48 and exceeding 30% by week 3 (Figure). Other Canadian surveillance data surpassed the national epidemic threshold of 5% several weeks later, in week 51 [1]. Among SPSN participants, most case viruses have been influenza A (609/647; 94%). Among 581 subtyped influenza A viruses, 399 (69%) were A(H1N1)pdm09.

**Figure:**
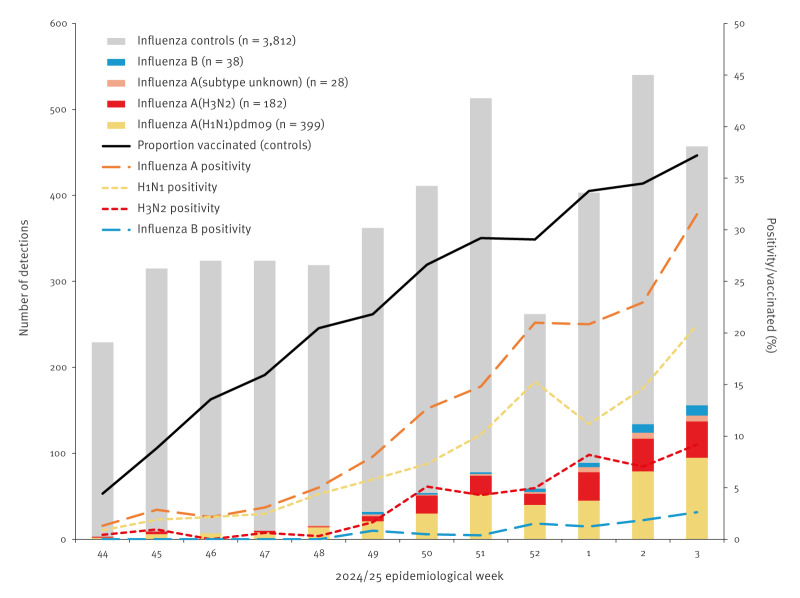
Influenza virus test-positive and test-negative specimens, by week of specimen collection, Canadian Sentinel Practitioner Surveillance Network, 27 October 2024–18 January 2025 (weeks 44–3) (n = 4,459)

The figure includes individuals missing comorbidity data, who are excluded from vaccine effectiveness (VE) analyses.

Displayed proportion vaccinated is based on those vaccinated ≥ 2 weeks before illness onset date.

Overall proportion vaccinated among influenza controls is 24% (903/3,812) when only considering those vaccinated ≥ 2 weeks before illness onset date. Without regard to vaccination timing, proportion vaccinated among influenza controls is 26% (1,030/3,939).

We sequenced more than one third of SPSN A(H1N1)pdm09 case viruses overall (142/399; 36%), with 31 (22%) belonging to clade 5a.2a.1 and 111 (78%) to clade 5a.2a, all of the latter being subclade C.1.9 defined by substitutions T120A, K169Q(Ca1), and I418V. Additional S83P and I510T substitutions further distinguish most of the C.1.9 viruses as C.1.9.3 (88/111; 79%). The genetic distribution of sequenced influenza A case viruses is provided in Supplementary Table S1. Of sequenced A(H1N1)pdm09 viruses, collection dates spanned epi-weeks 44–2, with increase in 5a.2a contribution from 66% (29/44) in epi-weeks 44–49 to 84% (82/98) in epi-weeks 50–2 (p = 0.018).

We sequenced nearly one third of SPSN A(H3N2) case viruses overall (54/182; 30%) between epi-weeks 44–2, with virtually all (52/54; 96%) belonging to clade 2a.3a.1. Among them, 46 of 52 (88%) belong to subclade J.2, defined by substitutions N122D(A)(-CHO) and K276E(C), with some having an additional S145N(A) substitution (10/46; 22%) and others having an additional T135K(A)(RBS)(-CHO) substitution (14/46; 30%). Of remaining 2a.3a.1 viruses, 6 of 52 (12%) instead belong to subclade J.2.2, defined by the additional S124N(A) substitution, of which most (4/6) also have the additional S145N(A) substitution.

## Epidemiological findings

Participant profiles including influenza A cases are displayed in Table 1 and stratified by subtype of case viruses in Supplementary Tables S2 and S3. Most participants overall were aged < 50 years old (67%), with similar median age between influenza A cases (35 years) and controls (37 years) (p = 0.263).

**Table 1:** Participant profile, influenza A analyses, Canadian Sentinel Practitioner Surveillance Network (SPSN), 27 October 2024–18 January 2025 (weeks 44–3) (n = 4,421)

Characteristics	All ARI participants (column %)						Influenza-vaccinated^a^ (row %)					
	Overall		Influenza A cases		Influenza controls		Overall		Influenza A cases^b^		Influenza controls^b^	
	n	%	n	%	n	%	n	%	n	%	n	%
N (row %)	4,421	100	609	14	3,812	86	1,004	23	101	17	903	24
**Age group (years)^c^**												
1–19	1,384	31	177	29	1,207	32	175	13	23	13	152	13
20–49	1,596	36	261	43	1,335	35	253	16	29	11	224	17
50–64	719	16	103	17	616	16	193	27	21	20	172	28
≥ 65	722	16	68	11	654	17	383	53	28	41	355	54
Median (IQR)	37 (13–57)		35 (15–52)		37 (13–57)		56 (34–71)		48 (25–66)		58 (34–72)	
**Sex**												
Female	2,641	60	358	59	2,283	60	639	24	67	19	572	25
Male	1,764	40	249	41	1,515	40	364	21	34	14	330	22
Unknown	16	0	2	0	14	0	1	6	0	0	1	7
**Comorbidity^d^**												
No	3,363	76	502	82	2,861	75	607	18	73	15	534	19
Yes	940	21	91	15	849	22	364	39	26	29	338	40
Unknown	118	3	16	3	102	3	33	28	2	13	31	30
**Province**												
Alberta	448	10	87	14	361	9	114	25	10	11	104	29
British Columbia	931	21	140	23	791	21	292	31	26	19	266	34
Ontario	1,868	42	325	53	1,543	40	456	24	59	18	397	26
Quebec	1,174	27	57	9	1,117	29	142	12	6	11	136	12
**Weeks of specimen collection, 2024/25^e^**												
44	229	5	3	0	226	6	10	4	0	0	10	4
45	315	7	9	1	306	8	28	9	1	11	27	9
46	324	7	7	1	317	8	43	13	0	0	43	14
47	324	7	10	2	314	8	52	16	2	20	50	16
48	319	7	16	3	303	8	64	20	2	13	62	20
49	359	8	29	5	330	9	76	21	4	14	72	22
50	409	9	52	9	357	9	102	25	7	13	95	27
51	511	12	76	12	435	11	137	27	10	13	127	29
52	258	6	55	9	203	5	69	27	10	18	59	29
1	398	9	84	14	314	8	123	31	17	20	106	34
2	530	12	124	20	406	11	159	30	19	15	140	34
3	445	10	144	24	301	8	141	32	29	20	112	37

ARI: acute respiratory illness; IQR: interquartile range.

Unless otherwise specified, values displayed in the columns represent the number of specimens per category and percentages are relative to the total.

^a^ Vaccination status based on participant or guardian report. Participants vaccinated < 2 weeks before onset of symptoms or with unknown vaccination status or timing were excluded.

^b^ Without regard to time before illness onset, 110 of 618 (18%) cases and 1,030 of 3,939 (26%) controls across the analysis period were vaccinated (p < 0.001).

^c^ Children < 1 year excluded as per usual in prior Canadian Sentinel Practitioner Surveillance Network (SPSN) analyses based on variability and/or uncertainty in their age-related vaccine eligibility over the course of the epidemic. Other age strata defined as per usual SPSN analyses predicated upon higher likelihood of chronic comorbidity at ≥ 50 years and higher age-associated risk among adults ≥ 65 years [25].

^d^ Includes chronic comorbidities that place individuals at higher risk of serious complications from influenza as defined by Canada’s National Advisory Committee on Immunization [25].

^e^ Missing specimen collection dates were imputed as the date the specimen was received and processed at the laboratory minus 2 days.

The percentage of specimens testing influenza A positive increased across the analysis period, concurrent with increasing vaccine coverage, while controls were more evenly distributed (Figure). Among adult controls aged ≥ 18 years presenting during the last epi-week of the study period, the proportion vaccinated regardless of timing before illness onset (100/228; 44%; data not shown) is comparable to the 2023/24 vaccine coverage survey among Canadian adults ≥ 18 years (42%) [11].

We estimated adjusted VE of 53% (95% CI: 36–65) against A(H1N1)pdm09 and 54% (95% CI: 29–70) against A(H3N2) (Table 2). The VE findings are comparable when excluding COVID-19 cases from influenza controls (≤ 4% difference), adjusting for sex (≤ 1% difference) or using Firth’s regression to address small sample size (≤ 1% difference) (data not shown). For both influenza A overall and A(H1N1)pdm09, age-stratified VE estimates are comparable if slightly higher in participants aged ≥ 65 vs < 65 years, with wide and overlapping confidence intervals.

**Table 2:** Vaccine effectiveness estimates against influenza A overall and by subtype, Canadian Sentinel Practitioner Surveillance Network (SPSN), 27 October 2024–18 January 2025 (weeks 44–3) (n = 4,303)

Influenza type or subtype	Total	Cases		Controls		VE^a,b^	
	N	n vac^c^/N	%	n vac^c^/N	%	%	95% CI
**Influenza A**	4,303	99/593	17	872/3,710	24	54	41–64
1–64 years	3,610	72/528	14	531/3,082	17	53	37–64
≥ 65 years	693	27/65	42	341/628	54	59	29–76
**Influenza A(H1N1)pdm09**	4,100	65/390	17	872/3,710	24	53	36–65
1–64 years	3,431	48/349	14	531/3,082	17	50	31–65
≥ 65 years	669	17/41	41	341/628	54	57	16–78
**Influenza A(H3N2)**	3,885	30/175	17	872/3,710	24	54	29–70

CI: confidence interval; vac: vaccinated; VE: vaccine effectiveness.

^a^ VE was calculated as 1 − odds ratios (OR)  x  100%. ORs compared test positivity between vaccinated and unvaccinated participants by logistic regression with covariate adjustment as specified. Unadjusted and adjusted ORs are presented in Supplementary Table S4. Firth’s logistic regression was explored to address small sample size but did not alter point estimates by more than 1% (absolute) (not displayed).

^b^ Adjusted for age group (1–19, 20–49, 50–64, ≥ 65 years), province (Alberta, BC, Ontario, Quebec), calendar time (bi-weekly epi-weeks 44–45, 46–47, 48–49, 50–51, 52–1, 2–3), and comorbidity (yes, no). With ≤ 3% of cases or controls overall (≤ 5% among ≥ 65 years) missing comorbidity information (vaccinated or unvaccinated), we excluded those with unknown comorbidity.

^c^ Vaccination status based upon participant or guardian report. Participants vaccinated < 2 weeks before acute respiratory illness onset or with unknown vaccine status or timing were excluded.

## Discussion

Despite a delayed start to the 2024/25 influenza season, the Canadian SPSN estimates that the influenza vaccine has approximately halved the risk of outpatient ARI among vaccinated vs unvaccinated individuals through mid-January 2025, with the vaccine protecting comparably well against A(H1N1)pdm09 and A(H3N2) subtypes.

The 2024/25 mid-season estimate of VE against influenza A(H1N1)pdm09 (53%) is within the range of historic SPSN estimates from the past decade (ranging ca 40–70% since 2014/15) [12], but lower than estimated mid-season for 2023/24 (63%) [8]. As in 2023/24, clades 5a.2a and 5a.2a.1 continue to co-circulate, the latter retained as 2024/25 vaccine strain. Thus far, more A(H1N1)pdm09 viruses characterised by the SPSN belong to the vaccine-mismatched clade 5a.2a in 2024/25 compared with 2023/24, i.e. 78% vs 49%, respectively, with the United States (US) (ca 60%) and Europe (ca 95%) also reporting relative 5a.2a predominance in 2024/25 [13,14]. Paradoxically, in 2023/24 the SPSN and networks elsewhere (US, Europe) reported higher VE against vaccine-mismatched clade 5a.2a viruses, for reasons the SPSN postulated in its 2023/24 mid-season publication, including mutations in the egg-adapted vaccine strain and/or imprint-related effects [8,15,16]. As such, the lower SPSN VE despite greater 5a.2a contribution in 2024/25 may be unexpected. We hope to explore these effects and the potential impact of repeat vaccination with unchanged A(H1N1)pdm09 antigen in end-of-season analyses [17,18].

To date, A(H3N2) viruses comprise less than one-third (31%) of influenza A viruses subtyped by the SPSN. Among primary care sentinel sites in Europe, about one quarter are A(H3N2) through week 3 of 2025 [14]. A second consecutive season of A(H1N1)pdm09 predominance in both Canada and Europe may be unexpected [8,19]. However, A(H3N2) has thus far contributed more in the US (ca 55%) and may also show a further increase in Canada and Europe through the remainder of the season [1,13,14]. In 2024/25, we report comparable VE against A(H3N2) and A(H1N1)pdm09, which is also unusual. As reported historically by the SPSN and others, A(H3N2) VE estimates often fall below 50% and are typically lower than A(H1N1)pdm09 [12,20]. Improved A(H3N2) VE this season (54%), compared with 2023/24 mid- and end-of-season estimates of the SPSN (40%) and other outpatient VE networks (30%) [8,12,15,16], may reflect an updated vaccine strain, now better clade-matched to 2a.3a.1 viruses that persist with little antigenic advance [21]. Our observation of adjacent substitutions S145N or T135K in antigenic site A close to the RBS of some 2a.3a.1 viruses, however, may warrant attention. S145N is an antigenic cluster transition site [22] that could contribute to antigenic drift among recently circulating viruses [23]. T135K constitutes loss of glycosylation at N133, which we have previously shown to be associated with lower VE point estimates [24]. Together with N122 loss of glycosylation characteristic of the J.2 subclade, this additional loss of glycosylation by the T135K subcluster could expose a region otherwise shielded by glycosylation since 1998 (when N133 and N122 were co-acquired). Potential impact on VE elsewhere and through the rest of the season requires further monitoring.

As for all observational studies, limitations include residual bias and confounding. We do not collect or adjust for socioeconomic status (SES) or race as potential confounders. These factors may be particularly relevant to cohort studies that compare disease incidence. Conversely, all participants in SPSN TND analyses have acquired ARI and accessed primary healthcare. In estimating VE, the TND then compares ARI participants who have influenza virus vs another aetiology for the ARI, upon which SES or race are less likely to exert differential influence. Low-level influenza associated with delayed season onset affects power and precision in this mid-season analysis and precludes extensive stratification. With ca 90% of influenza vaccines across SPSN provinces being egg-based and inactivated, product-specific comparisons are also precluded. Current estimates reflect protection up to 2–3 months post-vaccination; waning effects may be later explored as may also clade/subclade-specific analyses. In generalising findings, varying vaccination programs, subtype, and clade contributions should be considered.

## Conclusions

Interim estimates from the SPSN suggest the 2024/25 influenza vaccine reduces the risk of medically attended ARI due to influenza by about half among vaccinated vs unvaccinated individuals, similarly for A(H1N1)pdm09 and A(H3N2) subtypes. In a second consecutive season of A(H1N1)pdm09 predominance and A(H3N2) co-circulation, including unchanged A(H1N1)pdm09 but updated A(H3N2) vaccine antigens, multifactorial contributions to VE remain relevant pursuits for end-of-season analyses, e.g. prior vaccination, age/birth cohort, genetic and antigenic relatedness.

## Supplementary Data

308000 Supplement
