# Supplementary material for: Interim estimates of vaccine effectiveness against influenza A(H1N1)pdm09 and A(H3N2) during a delayed influenza season, Canada, 2024/25
Source: Euro Surveill. 2025 Jan 30;30(4):2500059. doi: 10.2807/1560-7917.ES.2025.30.4.2500059 (PMC11920781; doi:10.2807/1560-7917.ES.2025.30.4.2500059)
Supplement: Supplement [file 25-00059_SKOWRONSKI_Supplement.pdf]

Supplementary Materials

This supplementary material is hosted by *Eurosurveillance* as supporting information alongside the article “*Interim estimates of vaccine effectiveness against influenza A(H1N1)pdm09 and A(H3N2) during a delayed influenza season, Canada, 2024/25*” on behalf of the authors who remain responsible for the accuracy and appropriateness of the content. The same standards for ethics, copyright, attributions and permissions as for the article apply. Supplements are not edited by *Eurosurveillance* and the journal is not responsible for the maintenance of any links or email addresses provided therein.

Table of Contents (Hyperlinked)

Supplementary Table S1. Genetic distribution of Influenza A case viruses (n=581) by subgroup included in vaccine effectiveness analyses, Canadian Sentinel Practitioner Surveillance Network (SPSN), 27 October 2024 – 18 January 2025 (weeks 44–3). 3

Supplementary Table S2. Participant profile, influenza A(H1N1)pdm09 analyses, Canadian Sentinel Practitioner Surveillance Network (SPSN), 27 October 2024 – 18 January 2025 (weeks 44–3) (n = 4,211). 4

Supplementary Table S3. Participant profile, influenza A(H3N2) analyses, Canadian Sentinel Practitioner Surveillance Network (SPSN), 27 October 2024 – 18 January 2025 (weeks 44–3) (n = 3,994). 6

Supplementary Table S4. Odds ratio of association between vaccination and acute respiratory illness due to influenza, Canadian Sentinel Practitioner Surveillance Network (SPSN), 27 October 2024 – 18 January 2025 (weeks 44–3) (n = 4,303) 8

References, Supplementary Material 9

Supplementary Table S1. Genetic distribution of Influenza A case viruses (n=581) by subgroup included in vaccine effectiveness analyses, Canadian Sentinel Practitioner Surveillance Network (SPSN), 27 October 2024 – 18 January 2025 (weeks 44–3).

**Genetic clade, as defined by ECDC [1] based upon specific HA amino acid substitutions**[1,2]<br>+ additional substitutions by subclade or uniquely identified (antigenic site)	**NextStrain subclade** [3]	**BC**	**Alberta**	**Ontario**	**Québec**	**TOTAL**
**Influenza A(H1N1)pdm09, N (case viruses)**<br>Case viruses successfully sequenced, n (% n/N)		**81**<br>52 (64%)	**30**<br>11 (37%)	**252**<br>70 (28%)	**36**<br>9 (25%)	**399**<br>142 (36%)
**5a.2a = 5a.2 + K54Q + A186T (Sb) + Q189E (Sb) + E224A (RBS) + R259K + K308R**	**C.1**	**39**	**6**	**61**	**5**	**111**
+ T120A + K169Q (Ca1) + HA2: I91V	C.1.9	5		13	2	20
+ P137S (Ca2)	C.1.9.1	1				1
+ S83P + HA2: I183T		4		18	2	24
+ I166V (Ca1)	C.1.9.3	10	2	28	1	41
+ T216A[3]		19	4			23
+ Q54K + D86N + N125D (Sa) + I149V	C.1.9.4			2		2
**5a.2a.1 = 5a.2a + P137S (Ca2) + K142R (Ca2) + D260E + T277A + HA2: E29D + I91V + N124H**	**C.1.1**	**13**	**5**	**9**	**4**	**31**
+ T216A	D			3	1	4
+ T120A + HA2: I45V	D.3			2		2
+ R45K	D.5	13	5	4	3	25
**Influenza A(H3N2), N (case viruses)**<br>Case viruses successfully sequenced, n (% n/N)		**44**<br>29 (66%)	**56**<br>12 (21%)	**64**<br>7 (11%)	**18**<br>6 (33%)	**182**<br>54 (30%)
**2a.3a = 2a + D53N (C) + N96S (D)(+CHO) + I192F (B) + E50K (C) + HA2: N49S**	**G.1.3.1**				**2**	**2**
**2a.3a.1 = 2a.3a + I140K (A) + I223V**	**J**	**29**	**12**	**7**	**4**	**52**
+ N122D (A)(-CHO) + K276E (C)		10	9	2	1	22
+ S145N (A)	J.2	4	2	4		10
+ T135K (A)(RBS)(-CHO)		11	1		2	14
+ S124N (A)[4]	J.2.2	4		1	1	6

BC: British Columbia; ECDC: European Centre for Disease Control and Prevention; -CHO: loss of potential glycosylation site; RBS: receptor binding site.

[1] Influenza A(H1N1)pdm09 substitutions are relative to A/Wisconsin/588/2019 (EPI_ISL_404460) 5a.2 reference virus and Influenza A(H3N2) relative to A/Thailand/8/2022 (EPI_ISL_18399658) 2a.3a.1 reference virus [2]

[2] WGS performed using Oxford Nanopore technology with inclusion criteria of ≥50X depth of coverage across ≥90% of the HA segment

[3] Potential glycosylation impacts and imprint-related effects [4]

[4] Includes four BC viruses with additional S145N(A) substitution

Supplementary Table S2. Participant profile, influenza A(H1N1)pdm09 analyses, Canadian Sentinel Practitioner Surveillance Network (SPSN), 27 October 2024 – 18 January 2025 (weeks 44–3) (n = 4,211).

Characteristics	All ARI participants (column %)						Influenza vaccinated[a] (row %)					
	Overall		Influenza A(H1N1) cases		Influenza controls		Overall		Influenza A(H1N1) cases[b]		Influenza controls[b]	
	n	%	n	%	n	%	n	%	n	%	n	%
N (row %)	4211	100	399	9	3812	91	970	23	67	17	903	24
**Age group (years)[c]**												
1–19	1334	32	127	32	1207	32	169	13	17	13	152	13
20–49	1489	35	154	39	1335	35	242	16	18	12	224	17
50–64	690	16	74	19	616	16	186	27	14	19	172	28
≥ 65	698	17	44	11	654	17	373	53	18	41	355	54
Median (IQR)	37 (13-57)		39 (13-53)		37 (13-57)		56.5 (34-71)		48 (17-67)		58 (34-72)	
**Sex**												
Female	2518	60	235	59	2283	60	618	25	46	20	572	25
Male	1678	40	163	41	1515	40	351	21	21	13	330	22
Unknown	15	0	1	0	14	0	1	7	0	0	1	7
**Comorbidity[d]**												
No	3190	76	329	82	2861	75	581	18	47	14	534	19
Yes	910	22	61	15	849	22	356	39	18	30	338	40
Unknown	111	3	9	2	102	3	33	30	2	22	31	30
**Province**												
Alberta	391	9	30	8	361	9	108	28	4	13	104	29
British Columbia	872	21	81	20	791	21	284	33	18	22	266	34
Ontario	1795	43	252	63	1543	40	439	24	42	17	397	26
Quebec	1153	27	36	9	1117	29	139	12	3	8	136	12
**Weeks of specimen collection, 2024/25[e]**												
44	228	5	2	1	226	6	10	4	0	0	10	4
45	312	7	6	2	306	8	28	9	1	17	27	9
46	324	8	7	2	317	8	43	13	0	0	43	14
47	322	8	8	2	314	8	51	16	1	13	50	16
48	317	8	14	4	303	8	63	20	1	7	62	20
49	351	8	21	5	330	9	76	22	4	19	72	22
50	387	9	30	8	357	9	101	26	6	20	95	27
51	487	12	52	13	435	11	135	28	8	15	127	29
52	243	6	40	10	203	5	64	26	5	13	59	29
1	359	9	45	11	314	8	117	33	11	24	106	34
2	485	12	79	20	406	11	153	32	13	16	140	34
3	396	9	95	24	301	8	129	33	17	18	112	37

ARI: Acute respiratory illness; IQR: interquartile range. Unless otherwise specified, values displayed in the columns represent the number of specimens per category and percentages are relative to the total.

[a] Vaccination status based on participant or guardian report. Participants vaccinated < 2 weeks before onset of symptoms or with unknown vaccination status or timing were excluded.

[b] Without regard to time before illness onset, 71 of 403 (18%) cases and 1,030 of 3,939 (26%) controls across the analysis period were vaccinated (p<0.001).

[c] Children < 1 year excluded as per usual in prior SPSN analyses based on variability and/or uncertainty in their age-related vaccine

eligibility over the course of the epidemic. Other age strata defined as per usual SPSN analyses predicated upon higher likelihood of chronic comorbidity at ≥ 50 years; and higher age-associated risk among adults ≥ 65 years [5].

[d] Includes chronic comorbidities that place individuals at higher risk of serious complications from influenza as defined by Canada's National Advisory Committee on Immunization [5].

[e] Missing specimen collection dates were imputed as the date the specimen was received and processed at the laboratory minus 2 days.

Supplementary Table S3. Participant profile, influenza A(H3N2) analyses, Canadian Sentinel Practitioner Surveillance Network (SPSN), 27 October 2024 – 18 January 2025 (weeks 44–3) (n = 3,994).

Characteristics	All ARI participants (column %)						Influenza vaccinated[a] (row %)					
	Overall		Influenza A(H3N2) cases		Influenza controls		Overall		Influenza A(H3N2) cases[b]		Influenza controls[b]	
	n	%	n	%	n	%	n	%	n	%	n	%
N (row %)	3994	100	182	5	3812	95	933	23	30	16	903	24
**Age group (years)[c]**												
1–19	1254	31	47	26	1207	32	158	13	6	13	152	13
20–49	1424	36	89	49	1335	35	233	16	9	10	224	17
50–64	639	16	23	13	616	16	178	28	6	26	172	28
≥ 65	677	17	23	13	654	17	364	54	9	39	355	54
Median (IQR)	36 (13-57)		29.5 (19-50)		37 (13-57)		57 (34-71)		49.5 (25-66)		58 (34-72)	
**Sex**												
Female	2388	60	105	58	2283	60	590	25	18	17	572	25
Male	1591	40	76	42	1515	40	342	21	12	16	330	22
Unknown	15	0	1	1	14	0	1	7	0	0	1	7
**Comorbidity[d]**												
No	3008	75	147	81	2861	75	556	18	22	15	534	19
Yes	877	22	28	15	849	22	346	39	8	29	338	40
Unknown	109	3	7	4	102	3	31	28	0	0	31	30
**Province**												
Alberta	417	10	56	31	361	9	110	26	6	11	104	29
British Columbia	835	21	44	24	791	21	271	32	5	11	266	34
Ontario	1607	40	64	35	1543	40	413	26	16	25	397	26
Quebec	1135	28	18	10	1117	29	139	12	3	17	136	12
**Weeks of specimen collection, 2024/25[e]**												
44	227	6	1	1	226	6	10	4	0	0	10	4
45	309	8	3	2	306	8	27	9	0	0	27	9
46	317	8	0	0	317	8	43	14	0	0	43	14
47	316	8	2	1	314	8	51	16	1	50	50	16
48	304	8	1	1	303	8	63	21	1	100	62	20
49	336	8	6	3	330	9	72	21	0	0	72	22
50	378	9	21	12	357	9	96	25	1	5	95	27
51	457	11	22	12	435	11	129	28	2	9	127	29
52	216	5	13	7	203	5	64	30	5	38	59	29
1	347	9	33	18	314	8	112	32	6	18	106	34
2	444	11	38	21	406	11	146	33	6	16	140	34
3	343	9	42	23	301	8	120	35	8	19	112	37

ARI: Acute respiratory illness; IQR: interquartile range. Unless otherwise specified, values displayed in the columns represent the number of specimens per category and percentages are relative to the total.

[a] Vaccination status based on participant or guardian report. Participants vaccinated < 2 weeks before onset of symptoms or with unknown vaccination status or timing were excluded.

[b] Without regard to time before illness onset, 34 of 186 (18%) cases and 1,030 of 3,939 (26%) controls across the analysis period were vaccinated (p=0.017).

[c] Children < 1 year excluded as per usual in prior SPSN analyses based on variability and/or uncertainty in their age-related vaccine eligibility over the course of the epidemic. Other age strata defined as per usual SPSN analyses predicated upon higher likelihood of

chronic comorbidity at ≥ 50 years; and higher age-associated risk among adults ≥ 65 years [5].

[d] Includes chronic comorbidities that place individuals at higher risk of serious complications from influenza as defined by Canada's National Advisory Committee on Immunization [5].

[e] Missing specimen collection dates were imputed as the date the specimen was received and processed at the laboratory minus 2 days.

Supplementary Table S4. Odds ratio of association between vaccination and acute respiratory illness due to influenza, Canadian Sentinel Practitioner Surveillance Network (SPSN), 27 October 2024 – 18 January 2025 (weeks 44–3) (n = 4,303)

	Total	Cases		Controls		Unadjusted OR[a]		Adjusted OR[a,b]	
	N	n vac[c]/N	%	n vac[c]/N	%	OR	95% CI	OR	95% CI
**Influenza A**	4303	99/593	17	872/3710	24	0.65	0.52 to 0.82	0.46	0.36 to 0.59
1-64 years	3610	72/528	14	531/3082	17	0.76	0.58 to 0.99	0.47	0.36 to 0.63
≥ 65 years	693	27/65	42	341/628	54	0.60	0.36 to 1.00	0.41	0.24 to 0.71
**Influenza A(H1N1)pdm09**	4100	65/390	17	872/3710	24	0.65	0.49 to 0.86	0.47	0.35 to 0.64
1-64 years	3431	48/349	14	531/3082	17	0.77	0.56 to 1.05	0.50	0.35 to 0.69
≥ 65 years	669	17/41	41	341/628	54	0.60	0.31 to 1.13	0.43	0.22 to 0.84
**Influenza A(H3N2)**	3885	30/175	17	872/3710	24	0.67	0.45 to 1.00	0.46	0.30 to 0.71

OR: odds ratio; CI: confidence interval; vac: vaccinated.

[a] ORs compared test positivity between vaccinated and unvaccinated participants by logistic regression.

[b] Adjusted for age group (1–19, 20–49, 50–64, ≥ 65 years), province (Alberta, BC, Ontario, Quebec), calendar time (bi-weekly epi-weeks 44-45, 46-47, 48-49, 50-51, 52-1, 2-3), and comorbidity (yes, no). With ≤3% of cases or controls overall (≤5% among ≥65 years) missing comorbidity information (vaccinated or unvaccinated), we excluded those with unknown comorbidity.

[c] Vaccination status based upon participant or guardian report. Participants vaccinated <2 weeks before acute respiratory illness onset or with unknown vaccine status or timing were excluded.

References, Supplementary Material

1. European Centre for Disease Prevention and Control. Influenza virus characterization - Summary Europe, May 2024 [Internet]. Stockholm: ECDC; 2024 May. Available from: https://www.ecdc.europa.eu/en/publications-data/influenza-virus-characterization-summary-europe-may-2024

2. Shu Y, McCauley J. GISAID: Global initiative on sharing all influenza data – from vision to reality. Eurosurveillance [Internet]. 2017 Mar 30 [cited 2025 Jan 27];22(13). Available from: https://www.eurosurveillance.org/content/10.2807/1560-7917.ES.2017.22.13.30494

3. Aksamentov I, Roemer C, Hodcroft E, Neher R. Nextclade: clade assignment, mutation calling and quality control for viral genomes. J Open Source Softw. 2021 Nov 30;6(67):3773.

4. Skowronski DM, Zhan Y, Kaweski SE, Sabaiduc S, Khalid A, Olsha R, et al. 2023/24 mid-season influenza and Omicron XBB.1.5 vaccine effectiveness estimates from the Canadian Sentinel Practitioner Surveillance Network (SPSN). Eurosurveillance. 2024 Feb 15;29(7):1–12.

5. National Advisory Committee on Immunization (NACI). National Advisory Committee on Immunization (NACI) Statement on Seasonal Influenza Vaccine for 2024–2025 [Internet]. Ottawa: Public Health Agency of Canada; 2024 May. Available from: https://www.canada.ca/content/dam/phac-aspc/documents/services/publications/vaccines-immunization/national-advisory-committee-immunization-statement-seasonal-influenza-vaccine-2024-2025/naci-statement-2024-07-25.pdf
